# Pilot Study for the Dietary Assessment of Xenobiotics Derived from Food Processing in an Adult Spanish Sample

**DOI:** 10.3390/foods11030470

**Published:** 2022-02-05

**Authors:** Aida Zapico, Sergio Ruiz-Saavedra, María Gómez-Martín, Clara G. de los Reyes-Gavilán, Sonia González

**Affiliations:** 1 Department of Functional Biology, University of Oviedo, 33006 Oviedo, Spain; aiida.zapiico@gmail.com (A.Z.); ruizssergio@uniovi.es (S.R.-S.); gomezmarmaria@uniovi.es (M.G.-M.); 2 Diet, Microbiota and Health Group, Instituto de Investigación Sanitaria del Principado de Asturias (ISPA), 33011 Oviedo, Spain; greyes_gavilan@ipla.csic.es; 3 Department of Microbiology and Biochemistry of Dairy Products, Instituto de Productos Lácteos de Asturias (IPLA-CSIC), 33300 Villaviciosa, Spain

**Keywords:** diet, xenobiotics, heterocyclic amines, polycyclic aromatic hydrocarbons, nitrosamines, acrylamide, gastrointestinal health

## Abstract

Background: Although xenobiotics from food processing have gained support as possible drivers of the relationship between diet and some types of cancer, there are still few studies characterizing the intake of these compounds among different populations. Aim: To describe the intake of heterocyclic amines (HAs), polycyclic aromatic hydrocarbons (PAHs), nitrates, nitrites, nitrosamines, and acrylamide; and to identify dietary and lifestyle related factors. Methods: This was a descriptive cross-sectional study in 70 adult volunteers. Intake was registered by means of a food frequency questionnaire, including cooking methods, temperature, and degree of browning. The European Prospective Investigation into Cancer (EPIC) and the Computerized Heterocyclic Amines Resource for Research in Epidemiology of Disease (CHARRED) databases were used for xenobiotic estimation in conjunction with data from the European Food Safety Authority (EFSA) and U.S. Food and Drug Administration (FDA). Results: Dietary HAs (amino-alpha-carboline (AαC), 2-amino-3-methylimidazo (4,5,f) quinoline (IQ), 2-amino-3,8 dimethylimidazo (4,5,f) quinoxaline (MeIQx), 2-amino-3,4,8 trime-thylimidazo (4,5,f) quinoxaline (DiMeIQx), and 2-amino-1-methyl-6-phenylimidazo (4,5,b) pyridine (PhIP)) were mainly derived from meat and meat products, while benzo (a) pyrene (B(a)P), dibenzo (a) anthracene (DiB(a)A), and total PAHs were explained by oils and fats, alcoholic beverages, and milk, respectively. Microwaved, fried, grilled, broiled, barbecued, and braised cooking methods were mainly responsible for HAs and PAHs consumption. Conclusion: Based on the wide presence and levels of intake of these compounds in different sources, more efforts should be made to adjust their intake to the levels recommended by health agencies.

## 1. Introduction

Solid evidence supports the importance of environmental factors, with special focus on diet, in the development of different types of cancer [1,2]. Several hypotheses have been postulated to elucidate the possible mechanisms of this association. In general terms, red and processed meats have been identified as risk factors for cancer, contrary to what has been considered for plant-based foods, which seem to exhibit a protective effect [1,2,3]. Based on existing scientific evidence, the International Agency for Research on Cancer (IARC) evaluated red meat as “probably carcinogenic to humans” (Group 2A) and processed meat as “carcinogenic to humans” (Group 1) in 2015 [4]. However, in addition to the nutritional and bioactive compounds contained in these foods, different chemical substances could be incorporated as a consequence of the cooking, preservation, and processing performed to improve their digestibility, palatability, and safety [2]. From them, heterocyclic amines (HAs) and polycyclic aromatic hydrocarbons (PAHs), which are not naturally present in foodstuffs, but are formed during high-temperature cooking of foods, have been targeted as mediators of this relationship along with nitrites and nitrates, which are commonly used as food additives [1,5,6,7,8,9]. HAs are formed from muscle creatine and/or creatinine, sugars, and amino acids by the Maillard reaction [5]. Even though these elements are present at elevated levels in meat and fish muscle, the major dietary source is usually meat and meat products [10]. Another targeted compound, classified as a probable carcinogen to humans (Group 2A) by the IARC, is the acrylamide generated by the Maillard reaction in baked or fried carbohydrate-rich food sources, such as potatoes or cereals, by the condensation reaction between reducing sugars (glucose or fructose) and free amino acids (i.e., asparagine) [11,12].

Finally, PAHs are not usually present in raw foods, but have been reported in foods from industrialized areas as a result of the atmospheric contamination, exposure to which these products are subjected [13]. High levels of PAHs have been found in smoked products and grilled meats [5,7,14], formed by pyrolysis processes of organic matter at high temperatures; i.e., by direct contact of lipids with a flame or heat source, from smoke produced in cooking, or by incomplete combustion of wood or charcoal during the cooking process [14]. Once formed, these compounds are deposited on the meat being cooked [7].

All these chemical compounds with possible carcinogenic activity for humans are grouped under the denomination of xenobiotics; i.e., foreign substances that are not produced or are not found in the composition of the living organism [4]. In interpreting the scientific evidence between xenobiotics resulting from food processing and gastrointestinal health, several aspects should be considered; the risk of chronic dietary exposure to potentially carcinogenic compounds depends on the dose, frequencies, and combinations of xenobiotics being taken; the times of exposure to each compound, and the individual genetic susceptibilities. Then, differences in the way of recovering information, such as those regarding cooking questionnaires, the reference period of reported intake, and the use of different food composition databases, may at least partly explain the lack of consensus among studies [15,16]. Although no harmonized methods have been found in the literature, at the European level [17], when evaluating the dietary exposure to xenobiotics, the accurate assessment of individual food consumption is essential. The development of improved Food Frequency Questionnaires (FFQs) including cooking method, degree of doneness, and browning, apart from the traditional questions about food type, amount, and frequency of consumption, is necessary for nutritional assessment. Given that the intake of dietary xenobiotics may have an important impact on human health, our aim in the present work was to quantify their uptake in the population in order to define potential therapeutic targets, as well as to identify associations with other dietary components with which they may interact, increasing or reducing their genotoxic potential. This information could be useful to provide the basis for a more holistic view on the relationship between diet and health in the future.

## 2. Subjects and Methods

### 2.1. Sample Recruitment and Study Design

Recruitment of the sample was carried out by the nutrition group at the University of Oviedo by contacting individuals enrolled in the first semester of 2020/2021 at the University Program for Older Adults of the University of Oviedo (PUMUO) (*n* = 75). Eligibility criteria were to be over 50 years of age and not having been diagnosed with any digestive disorders. Those individuals interested in participating were informed of the objectives of the study and signed an informed consent form. Once the data were analyzed, all those who reported the existence of a major health condition (with the most frequent being cancer, Parkinson’s disease, or irritable bowel disease) or outlier daily intakes (energy intake below 1000 kcal/day or above 4000 kcal/day) were excluded.

This project was evaluated and approved by the Regional Ethics Committee of Clinical Research of Asturias (Ref. 163/19) and by the Committee on Bioethics of CSIC (Ref. 174/2020). The procedures were performed in accordance with the fundamental principles set out in the Declaration of Helsinki, the Oviedo Bioethics Convention, and the Council of Europe Convention on Human Rights and Biomedicine, as well as in Spanish legislation on bioethics. Directive 95/46/EC of the European Parliament and the Council of 24 October 1995, on the protection of individuals regarding the processing of personal data and on the free movement of such data, was strictly followed.

### 2.2. General Characteristics and Food Frequency Questionnaire (FFQ)

General characteristics of the questionnaire included information on age, nationality, gender, weight, height, educational level, and economic income, as well as questions related to lifestyle, physical activity, and gastrointestinal health, among others.

The auto-administered FFQ was constructed with 155 items and required an estimated duration of 30–45 min to be filled out. In addition to food and culinary preparations, the specific type of food was recorded, as well as cooking methods and other related questions, when necessary. For each food, the frequency of intake and portion size were registered by means of a validated photograph album adapted from the Pilot Study for Assessment of Nutrient intake and Food Consumption Among Kids in Europe (PANCAKE) study [18]. A specific section about cooking habits (boiled, fried, grilled, baked/broiled, or barbecued) and the degree of cooking or toasting in the case of meats, fried potatoes, or toasted bread (undercooked, medium, well-done, very well-done) were included in the FFQ. To standardize this point, photographs of the different temperatures, in which the degree of browning increased progressively, were developed specifically for this study: low, medium, well-done or very well-done were incorporated. Additionally, complementary questions such as which part of the food was consumed (breast or thigh in the case of chicken) or the possible consumption and/or cooking of the skin (cooking with skin and eating the skin; cooking with skin, but not consuming it; and cooking without skin) were incorporated in order to improve the quality of the information.

A 24 h dietary recall (R24h) was used to record the intake of each individual over the course of a day, as a method of validation of the FFQ, in a total of 39 participants. For this purpose, a survey was designed consisting of 14 questions in which the participant was asked to record in as much detail as possible everything consumed for breakfast, lunch, afternoon snack, and dinner. They were asked to specify the ingredients used in each preparation; the size of the portion; the type of food, if applicable; the possible accompaniment with drink or bread; the way the food was cooked; the cooking of the meats with or without skin; and the possible subsequent intake of the skin. Finally, the degree of toastiness of bread, French fries, and meat was collected by means of visual images. Spearman correlation analyses were conducted between the information obtained throughout the FFQ and R24h for the intake of the main xenobiotic compound; 71% of the xenobiotics studied showed significant Spearman correlations, ranging from r = 0.20 (2-amino-3,4 dimethylimidazo (4,5,f) quinoline ((MeIQ)) to r = 0.75 (combined nitroso compounds (Comb.)) (*p* < 0.05; data not shown). These values have been previously considered acceptable in the literature [19,20].

Body mass index (BMI) was calculated using the formula: weight (kg)/height (m)^2^. Subjects were classified in normal weight (18.5–24.9 kg/m^2^), overweight (25.0–29.9 kg/m^2^), and obese (≥30.0 kg/m^2^), based on the Spanish Society for the Study of Obesity (SEEDO) criteria [21].

### 2.3. Xenobiotic Estimation and Nutritional Analyses

Based on food consumption per individual, cooking method, cooking time, and degree of browning, the nutritional analysis of the sample was carried out. For this purpose, information on the consumption of HAs, PAHs, nitrates, and nitrites was obtained mainly from the European Prospective Investigation into Cancer and Nutrition (EPIC) Carcinogen Database [22]. The EPIC database compiles information obtained from 139 references regarding the content per 100 g of food in nitrosamines, HAs, PAHs, nitrites, and nitrates in more than 200 food items. The food composition table is classified according to the preservation method, cooking method, degree of browning, and temperature [22]. This information was provided by the Computerized Heterocyclic Amines Resource for Research in Epidemiology of Disease (CHARRED) database for those foods or culinary preparations not included in the EPIC database [23]. Acrylamide content was provided by the European Food Safety Authority (EFSA) categorization of European food products for monitoring purposes [24] and U.S. Food and Drug Administration (FDA) composition tables [25]. For each compound, the foods that accounted for at least 80% of its total intake were identified. The classification of the food groups was carried out according to the classification into 18 food groups of the Centre for Higher Education in Nutrition and Dietetics (CESNID) food composition tables [26]. For the meat and meat derivatives group, the IARC definition was used to break down the red meat and processed meat groups [4].

The analysis was completed with the CESNID [26] and the United States Department of Agriculture (USDA) food composition tables [27]. The polyphenol content of the foods was extracted from the Phenol Explorer (PHEX) database [28], and fiber content from the tables of Marlett and Cheung [29].

### 2.4. Digestive Function Self-Assessment Questionnaire

This questionnaire included some of the broader Rome III Criteria gastrointestinal functionality symptoms [30]. The 12 variables selected were: stomach pain, belching or reflux, heartburn, bloating, flatulencies, unpleasant taste in the mouth, nausea, bad breath, loss of appetite, abdominal pain, chest discomfort at night, and abdominal distention. Both the presence and intensity (from never to mild, moderate, severe, or very severe) of each of these symptoms were evaluated by the participant. The results obtained for each individual were represented by the percentage of symptoms presenting each of the intensities.

### 2.5. Statistical Analyses

Results were analyzed using the IBM SPSS software version 25.0 (IBM SPSS, Inc., Chicago, IL, USA) and RStudio software version 1.4.1103. Goodness of fit to the normal distribution was checked by means of the Kolmogorov–Smirnov test. As normality of the variables was not achieved, nonparametric tests were used. Overall, categorical variables were summarized as percentages and continuous ones using mean and standard deviations. *T*-test and Chi-squared analyses were performed for continuous and categorical variables, respectively (*p*-value < 0.05) with a Bonferroni correction. To deeper explore the associations between xenobiotics and dietary components, Spearman correlation analyses were conducted. A heatmap was generated using the RStudio software version 1.4.1103 package corrplot. GraphPad Prism 8 was used for graphical representations.

## 3. Results

### 3.1. Description of the Sample

A general description of general and health-related parameters is shown in Table 1. The sample had a mean age of 59 years with a BMI of 27 kg/m^2^, indicative of overweight. Concerning health-related parameters, most of the sample did not have a previous history of first- or second-degree colorectal cancer (CRC), and only around a 17% had asthma and/or allergies or hypertension, and 9% had diabetes. In relation to intestinal disorders (diarrhea, constipation, hemorrhoids, fissures, and fistulas or abscesses), statistically significant differences were detected according to gender in the percentage of hemorrhoids (higher in women) and in the absence of intestinal pathology (higher in men). The average stool frequency was once a day, and stool consistency was normal in most cases (60%). In addition, the proportion of individuals reporting the presence of bleeding (36%) was notable, albeit occasional (96%). The self-assessment of gastrointestinal functionality, adapted from the Rome III Criteria, showed that most subjects presented a moderate level of symptoms and an acceptable gastrointestinal health status.

The variation in the average daily intake of the different food groups according to gender is presented in Table 2. A higher intake of potatoes and tubers, alcoholic beverages, and other foods was observed in men.

### 3.2. Xenobiotics: Doses and Dietary Origin

With respect to the consumption of xenobiotics in the sample, no gender-specific statistically significant differences were found for any of the xenobiotic compounds considered (Supplementary Table S1). The average intake values for HAs, hydrocarbons, and acrylamide were within the range reported for the main sources of carcinogens, as can be seen in Table 3 [31,32,33,34,35,36,37].

**Figure 1:**
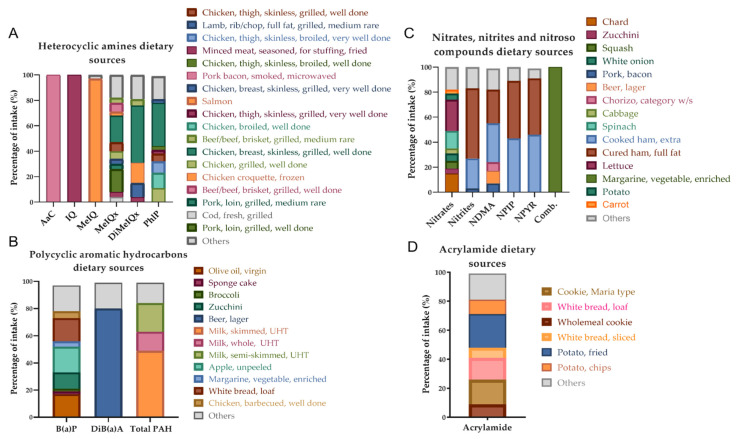
Main dietary sources of xenobiotics in the study sample. (**A**) Heterocyclic amine dietary sources. AαC, amino-alpha-carboline; IQ, 2-amino-3-methylimidazo (4,5,f) quinoline; MeIQ, 2-amino-3,4 dimethylimidazo (4,5,f) quinoline; MeIQx, 2-amino-3,8 dimethylimidazo (4,5,f) quinoxaline; DiMeIQx, 2-amino-3,4,8 trimethylimidazo (4,5,f) quinoxaline; PhIP, 2-amino-1-methyl-6-phenylimidazo (4,5,b) pyridine. (**B**) Polycyclic aromatic hydrocarbon dietary sources. B(a)P, benzo (a) pyrene; DiB(a)A, dibenzo (a) anthracene; Total PAHs, total polycyclic aromatic hydrocarbons. (**C**) Nitrate, nitrite, and nitroso compound dietary sources. NDMA, *N*-nitrosodimethylamine; NPIP, *N*-nitrosopiperidine; NPYR, *N*-nitrosopyrrolidine; Comb., combined nitroso compounds. (**D**) Acrylamide dietary sources.

According to our results, in the studied sample, dietary HAs (amino-alpha-carboline (AαC), 2-amino-3-methylimidazo (4,5,f) quinoline (IQ), 2-amino-3,8 dimethylimidazo (4,5,f) quinoxaline (MeIQx), 2-amino-3,4,8 trimethylimidazo (4,5,f) quinoxaline (DiMeIQx), and 2-amino-1-methyl-6-phenylimidazo (4,5,b) pyridine (PhIP)) were mainly derived from meat and meat products, with the exception of MeIQ, which was provided by fish (Figure 1). On the other hand, benzo (a) pyrene (B(a)P), dibenzo (a) anthracene (DiB(a)A), and total PAHs were more diversified in terms of dietary origin, being the main dietary sources oils and fats, alcoholic beverages, and milk, respectively. Nitrates derived predominantly from vegetables, while nitrites and *N*-nitroso compounds (NOCs) were mainly found in meat and meat products, and oils and fats. Acrylamide was provided at 64% by the group of cereals and derivatives. According to Figure 1, the main contributor to acrylamide intake was potato (33%) (fried potato and potato chips), followed by cookies (26%) (Maria-type cookies and whole meal cookies), and bread (22%) (loaf white bread and sliced white bread).

**Figure 2:**
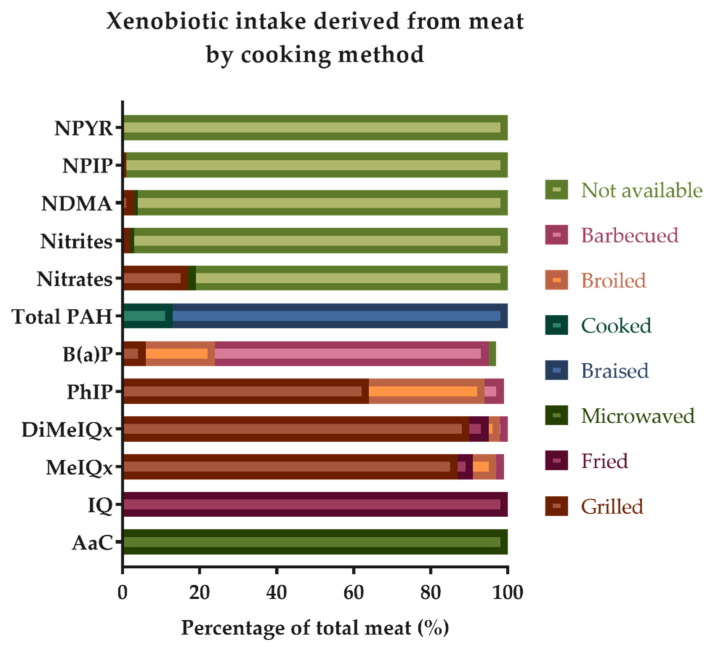
Impact of meat cooking method over the xenobiotic intake in the study sample (percentages may not sum to 100% because of rounding). The label “Cooked” was used by the authors Jakszyn, P. et al. as a general cooking method descriptor. AαC, amino-alpha-carboline; IQ, 2-amino-3-methylimidazo (4,5,f) quinoline; MeIQx, 2-amino-3,8 dimethylimidazo (4,5,f) quinoxaline; DiMeIQx, 2-amino-3,4,8 trimethylimidazo (4,5,f) quinoxaline; PhIP, 2-amino-1-methyl-6-phenylimidazo (4,5,b) pyridine; B(a)P, benzo (a) pyrene; DiB(a)A, dibenzo (a) anthracene; Total PAHs, total polycyclic aromatic hydrocarbons; NDMA, *N*-nitrosodimethylamine; NPIP, *N*-nitrosopiperidine; NPYR, *N*-nitrosopyrrolidine.

As depicted in Figure 2, microwaved, fried, grilled, broiled, barbecued, and braised cooking methods were mainly responsible for the intake of HAs and PAHs through the cooking of meat in the sample, whereas nitrates, nitrites, and nitrosamines (*N*-nitrosodimethylamine (NDMA), *N*-Nitrosopiperidine (NPIP), and *N*-Nitrosopyrrolidine (NPYR)) derived from grilled and other nonspecified methods. Processed meats were the main dietary source of these compounds (Figure 1), and they also contributed to the intake of hydrocarbons (B(a)P, not available) and amines (AαC and MeIQx, microwaved).

**Figure 3:**
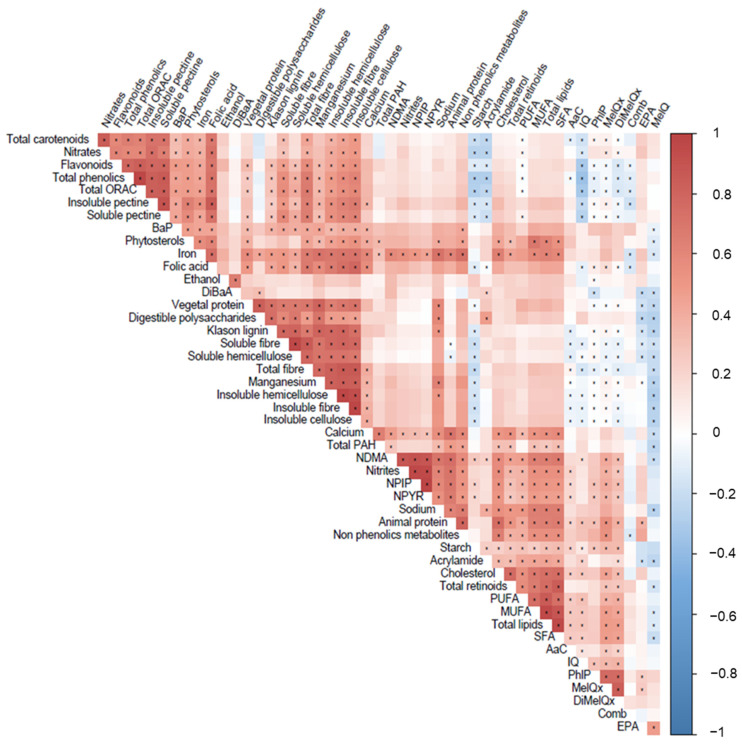
Graphical representation of correlations found between xenobiotic intake and other components derived from the diet. Only components showing significant correlations are represented. (*) *p*-value < 0.01. Total ORAC, total oxygen radical absorbance capacity; B(a)P, benzo (a) pyrene; DiB(a)A, dibenzo (a) anthracene; total PAHs, total polycyclic aromatic hydrocarbons; NDMA, *N*-nitrosodimethylamine; NPIP, *N*-nitrosopiperidine; NPYR, *N*-nitrosopyrrolidine; PUFA, polyunsaturated fatty acid; MUFA, monounsaturated fatty acid; SFA, saturated fatty acid; AαC, amino-alpha-carboline; IQ, 2-amino-3-methylimidazo (4,5,f) quinoline; PhIP, 2-amino-1-methyl-6-phenylimidazo (4,5,b) pyridine; MeIQx, 2-amino-3,8 dimethylimidazo (4,5,f) quinoxaline; DiMeIQx, 2-amino-3,4,8 trimethylimidazo (4,5,f) quinoxaline; Comb., combined nitroso compounds; EPA, eicosapentaenoic acid; MeIQ, 2-amino-3,4 dimethylimidazo (4,5,f) quinoline.

To further study the interaction between the consumption of xenobiotics and other dietary components, a correlation analysis was carried out (Figure 3). It was noteworthy that MeIQ was inversely related to elements of vegetable origin such as fiber (total and insoluble and soluble, insoluble cellulose, insoluble hemicellulose, soluble hemicellulose, and Klason lignin), other polysaccharides (starch and digestible polysaccharides), calcium, manganese, or sodium, among others; while other compounds such as IQ, from minced seasoned meat, were inversely related to the dietary total oxygen radical absorbance capacity (ORAC), as well as with compounds with high ORAC such as flavonoids, total phenolics, and insoluble and soluble pectin. Most of the xenobiotics quantified in the sample correlated significantly with the intake of cholesterol, total lipids, monounsaturated fatty acids (MUFA), polyunsaturated fatty acids (PUFA), saturated fatty acids (SFA), animal protein, iron, and sodium. When exploring the difference in the consumption of xenobiotics according to lifestyle and health-related gastrointestinal variables (Table 4), a higher mean consumption of nitrites, NDMA, NPIP, and NPYR in individuals who slept less than 7 h/day and in those who reported some occasional intestinal discomfort (such as hemorrhoids or fissures) was found. No significant differences were found according to smoking.

## 4. Discussion

The increasing and progressive incidence of some diseases such as cancer makes it urgent to develop adequate instruments for improving our understanding of the disease in order to increase the efficacy of medical treatments, but also for contributing to developing social guidelines to prevent the onset of the pathology. Diet is one of the modifiable lifestyle factors mainly contributing to the incidence and severity of some human pathologies [14]. As all dietary components and their interactions are important in the risk assessment, xenobiotic compounds formed during food cooking and processing have been targeted as mediators of the relationship between diet and cancer [1,3,4,5]. Overall, the comprehensive analyses carried out in this dietary study on an adult sample population enabled us to compare the intake of the main xenobiotics in our sample with that reported by other reference authors, and to specify their major dietary sources according to the cooking method. The identification of other dietary and lifestyle factors associated with the consumption of these compounds may be useful for the design of future studies attempting to understand their impact on health in more detail.

The HAs levels reported here were similar to those observed by other authors in different population groups with equivalent consumption of meat and meat products, vegetables, and fruits [32,38]. It should be noted that the dietary sources of some amines were less varied than those of other compounds in the same category. For example, 80% of the intake of MeIQx in the study sample was explained by 11 foods, followed by PhIP with eight and DiMeIQx with five. The best contributors to the intake of MeIQx, DiMeIQx, and PhIP were poultry meat (chicken, thigh, skinless, grilled, well done and very well-done; chicken, thigh, skinless, well-done; chicken, well-done; chicken, grilled, well-done), other animal meat sources (pork, grilled loin, well-done; beef/beef, brisket, grilled, medium-rare and well-done), or meat preparations (minced, seasoned, stuffing, fried, and chicken croquettes). In addition, MeIQx intake was also derived from the consumption of fish such as cod, fresh, grilled, and salmon. AαC and IQ amines were mainly supplied by animal foods, such as pork bacon, smoked, microwaved; and minced meat, seasoned for stuffing, fried, respectively. The 80% of DiB(a)A intake in the sample derived from lager beer, while milk, skimmed, UHT; milk, whole, UHT; milk, semi skimmed, UHT were the main dietary sources of total PAHs. The Comb. component was exclusively provided by vegetable, enriched margarine, while the nitrites NPIP and NPYR came mainly from processed meat products such as fatty cured ham and extra cooked ham. NDMA also came from other meats (chorizo, category w/s; and pork, bacon) (31%) and alcoholic beverages (beer, lager) (10%). Finally, nitrates were the compounds with the greatest variety of dietary sources. They were provided by vegetables, mainly lettuce, chard, and spinach.

The cooking methods of frying, grilling, barbecuing, microwaving, and stewing were mainly responsible for the consumption of HAs and PAHs from meats in the sample study, while some of these techniques, such as microwaving, are recognized as the lowest-driving xenobiotic-formation methods [7,39]. Nitrates were generated after grilling of meats and by other cooking methods that were not available in the database. These results were similar to the ones obtained by other authors [32,34,40]. Since for some references, the food composition table used had no information on the type of cooking, it was assumed that the resulting outcomes were dependent on the information available in the literature. For example, AαC was derived from a single food item (pork bacon, smoked, microwaved) that was always microwaved [41], while other compounds, such as DiMeIQx, appeared in the information for several food items, including chicken, breast, skinless with different cooking methods available (grilled, fried, broiled, and barbecued) [41,42].

In general, in our sample population, processed meats contributed mainly to the intake of nitrates, nitrites, and nitrosamines (NDMA, NPIP, and NPYR), although they also contributed to the intake of other compounds such as hydrocarbons (B(a)P) and HAs (AαC and MeIQx). Regarding meats, white meat was mainly consumed grilled (in Spain, this method implies the use of low amount of oil in a pan), while red meat was mostly cooked through frying (which implied food submerged in oil). As other authors have already pointed out, the cultural differences in the cooking methods employed are some of the main causes of variations in xenobiotic intake between populations [33]. On the other hand, the proportional contribution of white meat and red meat to the total intake of xenobiotics was similar, since, although the content of xenobiotics was lower in white meat, it was consumed more frequently and in greater daily quantities than red meat. These results may appear contradictory to current recommendations. However, it should be noted that there is scientific evidence showing that the potential carcinogenicity of red meat could be greater for the same intake of these xenobiotics, since another series of components such as heme groups or iron, which are found in higher levels in red meat, can promote endogenous nitrosation, which can contribute to an increased intake of xenobiotics by consumption of red meat [43]. Furthermore, other studies have found a link between proximal CRC and PhIP intake only when it came from red meat and not from white meat [6]. NDMA presented a higher intake level than the one reported in the literature [36], whereas no work estimating the intake of the rest of nitrosamines (NPIP, NPYR, and Comb) was available for comparison. The daily intake for nitrites (3 mg/day), although higher than the one reported by other authors (1 mg/day [36]), remained below the maximum intake recommended for an average weight of 75 kg (0.07 mg/kg body per day; 5 mg/day) [40], and the same applied to nitrates and acrylamide. These compounds showed mean daily intakes of 126 mg/day and 15 µg/day, respectively, which were lower than the maximum recommended intakes of 3.7 mg/kg body weight per day (278 mg/day) [44] and 0.17 mg/kg body weight per day (13 mg/day) [45] in each case. The main sources of acrylamide intake in our human sample were potato with 33% and bread with 22%, similar to previous studies in France and other European populations [11].

On the other hand, it was noteworthy that the direct associations reported between most of the main xenobiotic compounds and dietary compounds were from an animal origin, such as cholesterol, total lipids, MUFAs, PUFAs, SFAs, animal protein, iron, and sodium. These components were positively, and in most cases significantly, related to compounds belonging to the group of HAs. Nitrates, which mainly come from plant-based foods, have been directly related to compounds such as fiber and its subtypes (insoluble fiber, soluble fiber, insoluble cellulose, insoluble hemicellulose, insoluble pectin, soluble pectin), total carotenoids, total phenolics, flavonoids, or total ORAC, all of which have a proven beneficial impact on intestinal homeostasis preservation [14]. Thus, the upper limit of safety for the intake of xenobiotics may be conditioned by the subject’s antioxidant intake. In this regard, it has been shown that the intake of nitrates over 142.5 mg/day increases the risk of colon cancer only in those cases with a daily intake of vitamin C under 83.9 mg/day [46], and the intake of NDMA ≥ 0.07 μg/day was associated with an increased risk of this pathology with levels of vitamin E under the recommended amounts [47].

When comparing the HA intakes in our sample with the ones from other populations, it was noticeable that those studies from other countries in Europe showed lower amounts of MeIQx, DiMeIQx, PhIP, and total HAs consumption [31,33]. In Sweden, the calculation of individual mean PhIP intake still was maintained lower compared with the value in our sample (188 ng/day vs. 72 ng/day), but MeIQx and DiMeIQx intakes were higher (29 ng/day vs. 72 ng/day and 8 ng/day vs. 16 ng/day, respectively) [48]. Most of these European studies were related to the EPIC database, while studies from other continents were mainly based on the CHARRED database. In our study, we combined references from both databases. Indeed, when comparing with studies performed in the USA as a multiethnic cohort (MEC) study or from other countries such as Brazil, the value of total HAs and the quantified subclasses in those studies were higher than ours, except for DiMeIQx in MEC [15]. This could explain why we found values in between those of the European and non-European countries in our sample. The combination of both databases in order to obtain more standardized quantifications would be interesting in a more globalized and “diet-westernized” world, although xenobiotic formation is finally highly dependent on the culinary methods applied. As HAs have been highlighted as one of the responsible actors in the increasing CRC incidence, it is crucial to further elucidate how the quantities and the combinations of different HAs would impact on our health. For example, a meta-analysis performed in 2017 revealed an increase in the odds ratio (OR) for colorectal adenoma (CRA) risk of 1.26 for a 50 ng/day increment in MeIQx intake, but just an increase of 1.01 for a 100 ng/day increment in PhIP intake [5].

Regarding PAHs, the total amount recommended by the World Health Organization (WHO) ranges from less than 1 µg/day to 2 µg/day [49], and the Spanish Agency for Food Safety and Nutrition (AESAN) established the “No Observed Adverse Effect Level” (NOAEL) at 6.5 ng/kg/day per person [50], which for a 75 kg person would mean a maximum intake of 0.49 µg/day. In our sample, we found higher levels of total PAHs, although these values were in accordance with other studies performed previously in Spain [34,35].

The validation performed by an R24h showed an acceptable degree of accuracy in quantifying most of the xenobiotics in the diet; however, this observational study showed a few limitations. First, due to the high precision required for data collection, some differences between volunteers who regularly cooked and those who ate away from home might have occurred. Second, despite that the main strength of the study was the degree of detail in the questions and the use of photographic models, for the dietary collection of information, it represented an indirect estimation that was subjected to the systematic error inherent to this methodology. Third, quantification of the levels of these compounds in the organism would be desirable in the future as an additional validation step of the methodology applied. Finally, some recent publications demonstrated that some cooking methods such as air frying could reduce the formation of acrylamide and PAHs in comparison with deep-fat frying [51]. However, since this information was not available in the xenobiotics database, it was not considered.

## 5. Conclusions

Due to the wide presence of these compounds and their different sources, it is difficult to assess the impact of these dietary compounds on our health, but efforts should be made to adjust their intake to the levels recommended by health agencies.

In short, this preliminary exploratory study of the intake of dietary xenobiotics as potential carcinogens in a Spanish sample population can lay the foundation for short- and long-term broader and deeper multidisciplinary studies for the risk assessment of dietary exposure to these compounds and the onset of precancerous states.

## Figures and Tables

**Table 1:** General characteristics and description of gastrointestinal functionality, adapted from the Rome III Questionnaire, by gender.

Characteristics	Total (*N* = 70)	Gender	
		Male (*N* = 25)	Female (*N* = 45)
Age (years)	59 ± 12	62 ± 7	57 ± 14
<57	24 (34%)	5 (20%)	19 (42%)
57–65	18 (26%)	9 (36%)	9 (20%)
>66	28 (40%)	11 (44%)	17 (38%)
Energy intake (kcal/day)	1885.87 ± 581.71	1935.28 ± 569.40	1858.42 ± 593.01
Weight (kg)	74.70 ± 16.02	84.48 ± 16.84	69.15 ± 12.67 *
Height (m)	1.66 ± 0.08	1.74 ± 0.07	1.62 ± 0.05 *
BMI (kg/m^2^)	26.90 ± 4.64	27.77 ± 4.67	26.41 ± 4.60
Normal weight (18.5–24.9)	25 (36%)	7 (28%)	18 (40%)
Overweight (25.0–29.9)	32 (46%)	13 (52%)	19 (42%)
Obese (≥30.0)	12 (17%)	5 (20%)	7 (16%)
Na	1 (1%)	0 (0%)	1 (2%)
Smoking status			
Current smoker	7 (10%)	3 (12%)	4 (9%)
Former smoker	27 (39%)	14 (56%)	13 (29%) *
Never smoker	36 (51%)	8 (32%)	28 (62%) *
Exercise (hours/week)	1.13 ± 1.93	1.80 ± 2.20	0.76 ± 1.68 *
Sleeping (hours/day)	6.93 ± 1.11	6.80 ± 1.08	7.00 ± 1.13
Family CRC history			
Presence	11 (16%)	5 (20%)	6 (13%)
Absence	52 (74%)	18 (72%)	34 (76%)
Na	6 (9%)	2 (8%)	4 (9%)
Previous pathologies			
Hypertension	11 (16%)	7 (28%)	4 (9%) *
Diabetes	6 (9%)	3 (12%)	3 (7%)
Obesity	28 (40%)	12 (48%)	16 (36%)
Asthma and/or allergies	12 (17%)	4 (16%)	8 (18%)
None	14 (20%)	4 (16%)	10 (22%)
Intestinal pathologies			
Diarrhea	1 (1%)	0 (0%)	1 (2%)
Constipation	9 (13%)	1 (4%)	8 (18%)
Hemorrhoids	29 (41%)	6 (24%)	23 (51%) *
Fissures	2 (3%)	1 (4%)	1 (2%)
None	30 (43%)	17 (68%)	13 (29%) *
Bleeding frequency			
Daily	1 (1%)	1 (4%)	0 (0%)
At least once a week	0 (0%)	0 (0%)	0 (0%)
Occasionally	24 (34%)	8 (32%)	16 (36%)
Never	45 (64%)	16 (64%)	29 (64%)
Rome III Criteria			
No discomfort	49 ± 28	50 ± 24	49 ± 30
Mild discomfort	31 ± 21	33 ± 19	29 ± 22
Moderate discomfort	11 ± 12	8 ± 10	12 ± 14
Severe discomfort	1 ± 7	1 ± 2	2 ± 9
Very severe discomfort	1 ± 8	0 ± 2	2 ± 10
Na	7 ± 20	8 ± 23	7 ± 19
Stool frequency ^a^	7 ± 2	7 ± 2	7 ± 3
Stool consistency			
Liquid	0 (0%)	0 (0%)	0 (0%)
Soft	42 (60%)	15 (60%)	27 (60%)
Hard	27 (39%)	10 (40%)	17 (38%)

Values are presented as mean ± standard deviation or number of the subjects and percentage (%). CRC, colorectal cancer; Na, not available. (^a^) Number of depositions per week; (*) significant differences were found according to gender (*p*-value < 0.05).

**Table 2:** Differences in the intake of the major food groups in the study sample, by gender.

Food Groups Intake (g/Day)	Total (*N* = 70)	Gender	
		Male (*N* = 25)	Female (*N* = 45)
Cereals and cereals products	195.09 ± 138.37	185.08 ± 106.56	200.66 ± 154.09
Whole grain cereals	57.69 ± 118.62	23.31 ± 41.01	76.78 ± 141.78
Milk and dairy products	392.43 ± 236.26	323.14 ± 216.16	425.92 ± 242.56
Meat and meat products	147.47 ± 89.62	146.89 ± 72.32	147.79 ± 98.70
White meat	48.77 ± 37.88	48.05 ± 39.05	49.16 ± 37.66
Red meat	42.17 ± 30.04	47.13 ± 33.94	39.42 ± 27.66
Processed meat	58.90 ± 52.99	54.00 ± 28.47	61.62 ± 62.77
Eggs	43.51 ± 29.53	49.23 ± 33.74	40.33 ± 26.79
Fish	61.83 ± 36.99	63.46 ± 30.00	60.93 ± 40.66
Seafood	22.82 ± 19.64	22.92 ± 19.16	22.77 ± 20.12
Oils and fats	16.18 ± 8.57	18.05 ± 9.09	15.15 ± 8.19
Vegetables	308.53 ± 179.13	262.94 ± 153.23	333.86 ± 188.88
Legumes	42.61 ± 76.11	49.79 ± 77.89	38.62 ± 75.70
Potatoes and tubers	50.38 ± 31.75	60.50 ± 32.11	44.76 ± 30.46 *
Fruits	130.68 ± 90.87	156.27 ± 126.20	116.47 ± 60.69
Nuts and seeds	13.29 ± 17.60	9.12 ± 9.00	15.61 ± 20.65
Sugar and sweets	7.45 ± 10.11	9.93 ± 12.44	6.07 ± 8.39
Snacks	2.09 ± 4.45	3.16 ± 4.55	1.49 ± 4.32
Sauces and condiments	8.17 ± 7.17	8.04 ± 5.25	8.24 ± 8.10
Other foods	10.20 ± 14.37	14.84 ± 19.64	7.62 ± 9.72 *
Nonalcoholic beverages (mL/day)	225.86 ± 231.79	283.30 ± 325.24	193.96 ± 153.74
Alcoholic beverages (mL/day)	133.42 ± 171.11	191.02 ± 175.93	101.42 ± 161.55 *

Values are presented as mean ± standard deviation. (*) Significant differences were found between genders (*p*-value < 0.05).

**Table 3:** Comparison between mean xenobiotic intake in the study sample with other studies using EPIC and CHARRED databases.

Xenobiotics	Value (*N* = 70)	Type of Study					
		Reference Value	Sample Size (Gender)	Age (Years)	Health Status	Country	Reference
Heterocyclic amines (ng/day)							
MeIQx	29.48 ± 27.85	16.8 (±29.7)	*n* = 3.699 (MF)	35–65	Healthy	DE	[31] ^a^
		102.7	*n* = 561 (MF)	>20	Na	BR	[32] ^b^
DiMeIQx	8.18 ± 7.96	3.0 (±4.5)	*n* = 3.699 (MF)	35–65	Healthy	DE	[31] ^a^
		9.8	*n* = 561 (MF)	>20	Na	BR	[32] ^b^
PhIP	187.59 ± 257.04	41.0 (±117.5)	*n* = 3.699 (MF)	35–65	Healthy	DE	[31] ^a^
		324.3	*n* = 561 (MF)	>20	Na	BR	[32] ^b^
Total HAs	226.99 ± 285.50	69.4	*n* = 21.462 (MF)	35–65	Na	DE	[33] ^a^
		436.8	*n* = 561 (MF)	>20	Na	BR	[32] ^b^
Polycyclic aromatic hydrocarbons (µg/day)							
B(a)P	0.03 ± 0.03	0.14 (±0.07)	*n* = 40.690 (MF)	35–64	Na	SP	[34] ^a^
DiB(a)A	0.07 ± 0.10	0.06	*n* = 3.890.240 (M)	20–65	Na	SP	[35] ^c^
Total PAHs	5.04 ± 3.84	8.57 (±2.69)	*n* = 40.690 (MF)	35–64	Na	SP	[34] ^a^
Nitrates, nitrites, and nitroso compounds							
Nitrites (mg/day)	3.14 ± 2.90	1.48 (±0.51)	*n* = 20.095 (MF)	40–79	Healthy	UK	[36] ^a^
NDMA (µg/day)	0.17 ± 0.14	0.06 (±0.05)	*n* = 20.095 (MF)	40–79	Healthy	UK	[36] ^a^
NPIP (µg/day)	0.09 ± 0.09	72.3 (±19.2) ^d^	*n* = 20.095 (MF)	40–79	Healthy	UK	[36] ^a^
NPYR (µg/day)	0.15 ± 0.16						
Comb. (ng/day)	1.71 ± 5.10						
Acrylamide (µg/day)	15.12 ± 11.60	20.6 (±12.1)	*n* = 22.783 (F)	29–69	Cases & healthy	SP	[37] ^a^

Values are presented as mean ± standard deviation. (^a^) Study/data from the European Prospective Investigation on Cancer (EPIC); (^b^) study from the Computerized Heterocyclic Amines Resource for Research in Epidemiology of Disease (CHARRED); (^c^) study not belonging to either EPIC nor CHARRED, for which sample size was calculated using the National Statistics Institute (Spanish Statistics Office, available at: https://www.ine.es/en) to date (12 January 2021); (^d^) sum of all nitrosamines formed endogenously such as NPIP, NPYR, and Comb. MF, male and female; M, male; F, female; MeIQx, 2-amino-3,8 dimethylimidazo (4,5,f) quinoxaline; DiMeIQx, 2-amino-3,4,8 trimethylimidazo (4,5,f) quinoxaline; PhIP, 2-amino-1-methyl-6-phenylimidazo (4,5,b) pyridine; Total HAs, total heterocyclic amines; B(a)P, benzo (a) pyrene; DiB(a)A, dibenzo (a) anthracene; Total PAHs, total polycyclic aromatic hydrocarbons; NDMA, *N*-nitrosodimethylamine; NPIP, *N*-nitrosopiperidine; NPYR, *N*-nitrosopyrrolidine; Comb., combined nitroso compounds.

**Table 4:** Mean xenobiotic intake according to health characteristics.

Mean Daily Intake	Heterocyclic Amines (ng/Day)						Polycyclic Aromatic Hydrocarbons (μg/Day)			Nitroso Compounds (μg/Day)						Acrylamide (μg/Day)
	AαC	IQ	MeIQ	MeIQx	DiMeIQx	PhIP	B(a)P	DiB(a)A	Total PAHs	Nitrates (mg/Day)	Nitrites (mg/Day)	NDMA	NPIP	NPYR	Comb (ng/Day)	Acrylamide
BMI (kg/m^2^)																
Normal weight	0.01	0.15	1.79	27.92	7.88	159.69	0.03	0.06	3.89	118.39	2.85	0.16	0.08	0.14	2.80	11.96
Overweight	0.03	0.11	1.48	27.24	7.26	152.55	0.03	0.07	4.88	123.03	3.43	0.18	0.10	0.16	1.25	16.36
Obese	0.00	0.18	1.45	37.48	9.97	330.26	0.04	0.07	7.95 *	153.24	2.91	0.16	0.08	0.13	0.83	16.17
Smoking status																
Current smoker	0.01	0.15	1.95	35.17	10.32	177.52	0.04	0.04	4.35	110.49	2.41	0.13	0.07	0.12	1.43	21.37
Former smoker	0.01	0.14	1.46	25.88	7.57	226.07	0.03	0.09	4.82	130.36	2.73	0.14	0.07	0.11	1.11	12.16
Never smoker	0.03	0.14	1.60	31.09	8.21	160.69	0.03	0.05	5.35	125.77	3.58	0.19	0.11	0.18	2.22	16.13
Exercise																
Active	0.01	0.13	1.54	29.17	9.05	210.78	0.03	0.06	3.65	110.16	2.31	0.14	0.06	0.09	1.20	15.09
Sedentary	0.02	0.14	1.60	29.66	7.69	174.71	0.03	0.07	5.82 *	134.82	3.59	0.19	0.10 *	0.18 *	2.00	15.15
Sleeping																
≥7 h/day	0.02	0.13	1.47	27.88	7.24	185.21	0.03	0.06	4.61	130.64	2.65	0.14	0.07	0.12	1.20	13.38
<7 h/day	0.02	0.15	1.86	33.51	10.51	193.55	0.03	0.09	6.14	114.43	4.35 *	0.23 *	0.13 *	0.21 *	3.00	19.50 *
Intestinal pathologies																
Constipation	0.00	0.19	2.17	38.64	11.05	261.38	0.05	0.02	8.33	94.33	3.24	0.14	0.09	0.15	1.11	9.83
Regular transit	0.02	0.13	1.49	28.13	7.75	176.71	0.03	0.07	4.56 *	130.68	3.12	0.17	0.09	0.15	1.80	15.91
Hemorrhoids	0.03	0.15	1.47	33.13	9.70	195.17	0.03	0.07	4.83	126.60	4.01	0.22	0.12	0.20	0.34	16.67
No hemorrhoids	0.01	0.13	1.66	26.90	7.10	182.23	0.03	0.06	5.19	125.59	2.52 *	0.13 *	0.06 *	0.11 *	2.68	14.03
Fissures	0.05	0.40	2.73	36.88	10.96	469.58	0.02	0.04	2.72	120.76	7.87	0.37	0.24	0.39	5.00	16.54
No fissures	0.02	0.13 *	1.55	29.27	8.09	179.30	0.03	0.07	5.11	126.17	3.00 *	0.16 *	0.08 *	0.14 *	1.62	15.08
Bleeding																
Ever	0.03	0.14	1.43	28.74	8.94	180.16	0.03	0.09	4.76	124.45	3.96	0.22	0.12	0.20	0.80	15.21
Never	0.01	0.14	1.66	29.90	7.75	191.72	0.03	0.05	5.20	126.88	2.67	0.14 *	0.07	0.12	2.22	15.08
Rome III Criteria																
Moderate or greater ^a^	0.00	0.20	1.89	36.92	19.70	258.23	0.03	0.01	10.56	82.01	0.54	0.03	0.01	0.02	5.00	10.56
Never or mild ^b^	0.02	0.14	1.57	29.27	7.84 *	185.51	0.03	0.07	4.88 *	127.31	3.21	0.17	0.09	0.15	1.62	15.26

(^a^) Moderate or greater discomfort for more than 50% of the symptoms; (^b^) no discomfort or mild for a maximum of 50% of the symptoms; (*) significant differences were found between values belonging to the same category (*p*-value < 0.05). AαC, amino-alpha-carboline; IQ, 2-amino-3-methylimidazo (4,5,f) quinoline; MeIQ, 2-amino-3.4 dimethylimidazo (4,5,f) quinoline; MeIQx, 2-amino-3,8 dimethylimidazo (4,5,f) quinoxaline; DiMeIQx, 2-amino-3,4,8 trimethylimidazo (4,5,f) quinoxaline; PhIP, 2-amino-1-methyl-6-phenylimidazo (4,5,b) pyridine; B(a)P, benzo (a) pyrene; DiB(a)A, dibenzo (a) anthracene; Total PAHs, total polycyclic aromatic hydrocarbons; NDMA, *N*-nitrosodimethylamine; NPIP, *N*-nitrosopiperidine; NPYR, *N*-nitrosopyrrolidine; Comb., combined nitroso compounds.

## Data Availability

Not applicable.

## Supplementary Materials

The following are available online at https://www.mdpi.com/article/10.3390/foods11030470/s1, Table S1: Description of mean daily xenobiotic intake in the study sample, by gender.

## Abbreviations

AESAN	Spanish Agency for Food Safety and Nutrition
AαC	Amino-alpha-carboline
B(a)P	Benzo (a) pyrene
BMI	Body mass index
CESNID	Centre for Higher Education in Nutrition and Dietetics
CHARRED	Computerized Heterocyclic Amines Resource for Research in Epidemiology of Disease
Comb.	Combined nitroso compounds
CRA	Colorectal adenoma
CRC	Colorectal cancer
DiB(a)A	Dibenzo (a) anthracene
DiMeIQx	2-Amino-3,4,8 trimethylimidazo (4,5,f) quinoxaline
EFSA	European Food Safety Authority
EPIC European	Prospective Investigation into Cancer and Nutrition
FDA	U.S. Food and Drug Administration
FFQ	Food Frequency Questionnaire
HAs	Heterocyclic amines
IARC	International Agency for Research on Cancer
IQ	2-Amino-3-methylimidazo (4,5,f) quinoline
MEC	Multiethnic cohort
MeIQ	2-Amino-3.4 dimethylimidazo (4,5,f) quinoline
MeIQx	2-Amino-3,8 dimethylimidazo (4,5,f) quinoxaline
MUFA	Monounsaturated fatty acid
NDMA	*N*-nitrosodimethylamine
NOAEL	No observed adverse effect level
NOCs	*N*-Nitroso compounds
NPIP	*N*-Nitrosopiperidine
NPYR	*N*-Nitrosopyrrolidine
OR	Odds ratio
ORAC	Oxygen radical absorbance capacity
PAHs	Polycyclic aromatic hydrocarbons
PANCAKE	Assessment of Nutrient Intake and Food Consumption Among Kids in Europe
PHEX	Phenol Explorer
PhIP	2-Amino-1-methyl-6-phenylimidazo (4,5,b) pyridine
PUFA	Polyunsaturated fatty acid
PUMUO	University Program for Older Adults of the University of Oviedo
R24h	24-h dietary recall
SEEDO	Spanish Society for the Study of Obesity
SFA	Saturated fatty acid
USDA	United States Department of Agriculture
WHO	World Health Organization

## References

[1] De Kok T.M.C.M., Van Maanen J.M.S. (2000). Evaluation of fecal mutagenicity and colorectal cancer risk. Mutat. Res.-Rev. Mutat. Res..

[2] Nadeem H.R., Akhtar S., Ismail T., Sestili P., Lorenzo J.M., Ranjha M.M.A.N., Jooste L., Hano C., Aadil R.M. (2021). Heterocyclic aromatic amines in meat: Formation, isolation, risk assessment, and inhibitory effect of plant extracts. Foods.

[3] Etemadi A., Abnet C.C., Graubard B.I., Beane-Freeman L., Freedman N.D., Liao L., Dawsey S.M., Sinha R. (2018). Anatomical subsite can modify the association between meat and meat compounds and risk of colorectal adenocarcinoma: Findings from three large US cohorts. Int. J. Cancer.

[4] International Agency for Research on Cancer (IARC) Working Group (2018). Red Meat and Processed Meat: IARC Monographs on the Evaluation of Carcinogenic Risks to Humans.

[5] Chiavarini M., Bertarelli G., Minelli L., Fabiani R. (2017). Dietary intake of meat cooking-related mutagens (HCAs) and risk of colorectal adenoma and cancer: A systematic review and meta-analysis. Nutrients.

[6] Le N.T., Silva Michels F.A., Song M., Zhang X., Bernstein A.M., Giovannucci E.L., Fuchs C.S., Ogino S., Chan A.T., Sinha R. (2016). A prospective analysis of meat mutagens and colorectal cancer in the Nurses’ Health Study and Health Professionals Follow-up Study. Environ. Health Perspect..

[7] Miller P.E., Lazarus P., Lesko S.M., Cross A.J., Sinha R., Laio J., Zhu J., Harper G., Muscat J.E., Hartman T.J. (2013). Meat-related compounds and colorectal cancer risk by anatomical subsite. Nutr. Cancer.

[8] World Cancer Research Fund International, American Institute for Cancer Research (2018). Diet, Nutrition, Physical Activity and Colorectal Cancer.

[9] Jakszyn P., Agudo A., Ibãñez R., García-Closas R., Pera G., Amiano P., González C.A. (2004). Development of a food database of nitrosamines, heterocyclic amines, and polycyclic aromatic hydrocarbons. J. Nutr..

[10] Jahurul M.H.A., Jinap S., Ang S.J., Abdul-Hamid A., Hajeb P., Lioe H.N., Zaidul I.S.M. (2010). Dietary exposure to heterocyclic amines in high-temperature cooked meat and fish in Malaysia. Food Addit. Contam.-Part A Chem. Anal. Control Expo. Risk Assess..

[11] Dybing E., Farmer P.B., Andersen M., Fennell T.R., Lalljie S.P.D., Müller D.J.G., Olin S., Petersen B.J., Schlatter J., Scholz G. (2005). Human exposure and internal dose assessments of acrylamide in food. Food Chem. Toxicol..

[12] Mucci L.A., Adami H.O., Wolk A. (2006). Prospective study of dietary acrylamide and risk of colorectal cancer among women. Int. J. Cancer.

[13] Scientific Committee on Food Annex (2002). Background Document to the Opinion of the Scientific Committee on Food on the Risks to Human Health of Polycyclic Aromatic Hydrocarbons in Food (Expressed on 4 December 2002).

[14] Nogacka A.M., Gómez-Martín M., Suárez A., González-Bernardo O., de los Reyes-Gavilán C.G., González S. (2019). Xenobiotics formed during food processing: Their relation with the intestinal microbiota and colorectal cancer. Int. J. Mol. Sci..

[15] Ollberding N.J., Wilkens L.R., Henderson B.E., Kolonel L.N., Le Marchand L. (2012). Meat consumption, heterocyclic amines and colorectal cancer risk: The Multiethnic Cohort Study. Int. J. Cancer.

[16] Ritter-Gooder P.K., Lewis N.M., Heidal K.B., Eskridge K.M. (2006). Validity and reliability of a quantitative Food Frequency Questionnaire measuring n-3 fatty acid intakes in cardiac patients in the midwest: A validation pilot study. J. Am. Diet. Assoc..

[17] Sierra-Ruelas É., Bernal-Orozco M.F., Marcedo-Ojeda G., Márquez-Sandoval Y.F., Altamirano-Martínez M.B., Vizmanos B. (2020). Validation of semiquantitative FFQ administered to adults: A systematic review. Public Health Nutr..

[18] Ocké M., de Boer E., Brants H., van der Laan J., Niekerk M., van Rossum C., Temme L., Freisling H., Nicolas G., Casagrande C. (2012). PANCAKE—Pilot study for the assessment of nutrient intake and food consumption among kids in Europe. EFSA Support. Publ..

[19] Watson E.O., Heath A.L.M., Taylor R.W., Mills V.C., Barris A.C., Skidmore P.M.L. (2015). Relative validity and reproducibility of an FFQ to determine nutrient intakes of New Zealand toddlers aged 12–24 months. Public Health Nutr..

[20] Zazpe I., Santiago S., de la Pascual O.V., Romanos-Nanclares A., Rico-Campà A., Álvarez-Zallo N., Martínez-González M.Á., Martín-Calvo N. (2020). Validity and reproducibility of a semi-quantitative food frequency questionnaire in Spanish preschoolers—The Sendo Project. Nutr. Hosp..

[21] Foz M., Barbany M., Remesar X., Carrillo M., Aranceta J., García-Luna P., Alemany M., Vázquez C., Palou A., Sociedad Española para el Estudio de la Obesidad (SEEDO) (2000). Consenso SEEDO’ 2000 para la evaluación del sobrepeso y la obesidad y el establecimiento de criterios de intervención terapéutica. Med. Clin..

[22] Jakszyn P., Ibáñez R., Pera G., Agudo A., García-Closas R., Amiano P., González C.A. (2004). Food Content of Potential Carcinogens.

[23] National Institutes of Health (NIH) CHARRED: Computerized Heterocyclic Amines Resource for Research in Epidemiology of Disease. https://dceg.cancer.gov/tools/design/charred.

[24] European Food Safety Authority (EFSA) (2012). Update on acrylamide levels in food from monitoring years 2007 to 2010. EFSA J..

[25] Food and Drug Administration (FDA) (2015). Survey Data on Acrylamide in Food.

[26] Farran A., Zamora R., Cervera P. (2003). Tablas de Composición de Alimentos del Centro de Enseñanza Superior en Nutrición y Dietética (CESNID).

[27] United States Department of Agriculture (USDA) Food Composition Databases. https://ndb.nal.usda.gov/ndb/.

[28] Neveu V., Perez-Jiménez J., Vos F., Crespy V., du Chaffaut L., Mennen L., Knox C., Eisner R., Cruz J., Wishart D. (2010). Phenol-Explorer: An online comprehensive database on polyphenol contents in foods. Database.

[29] Marlett J., Cheung T. (1997). Database and quick methods of assessing typical dietary fiber intakes using data for 228 commonly consumed foods. J. Am. Diet. Assoc..

[30] Drossman D.A. (2006). The functional gastrointestinal disorders and the Rome III process. Gastroenterology.

[31] Rohrmann S., Hermann S., Linseisen J. (2009). Heterocyclic aromatic amine intake increases colorectal adenoma risk: Findings from a prospective European cohort study. Am. J. Clin. Nutr..

[32] Carvalho A.M., Miranda A.M., Santos F.A., Loureiro A.P.M., Fisberg R.M., Marchioni D.M. (2015). High intake of heterocyclic amines from meat is associated with oxidative stress. Br. J. Nutr..

[33] Rohrmann S., Zoller D., Hermann S., Linseisen J. (2007). Intake of heterocyclic aromatic amines from meat in the European Prospective Investigation into Cancer and Nutrition (EPIC)-Heidelberg cohort. Br. J. Nutr..

[34] Ibáñez R., Agudo A., Berenguer A., Jakszyn P., Tormo M.J., Sanchéz M.J., Quirós J.R., Pera G., Navarro C., Martinez C. (2005). Dietary intake of polycyclic aromatic hydrocarbons in a Spanish population. J. Food Prot..

[35] Falcó G., Domingo J.L., Llobet J.M., Teixidó A., Casas C., Müller L. (2003). Polycyclic Aromatic Hydrocarbons in foods: Human exposure through the diet in Catalonia, Spain. J. Food Prot..

[36] Loh Y.H., Jakszyn P., Luben R.N., Mulligan A.A., Mitrou P.N., Khaw K.T. (2011). N-nitroso compounds and cancer incidence: The European Prospective Investigation into Cancer and Nutrition (EPIC)-Norfolk Study. Am. J. Clin. Nutr..

[37] Obón-Santacana M., Kaaks R., Slimani N., Lujan-Barroso L., Freisling H., Ferrari P., Dossus L., Chabbert-Buffet N., Baglietto L., Fortner R.T. (2014). Dietary intake of acrylamide and endometrial cancer risk in the European Prospective Investigation into Cancer and Nutrition cohort. Br. J. Cancer.

[38] Wie G.A., Cho Y.A., Kang H.H., Ryu K.A., Yoo M.K., Kim Y.A., Jung K.W., Kim J., Lee J.H., Joung H. (2014). Red meat consumption is associated with an increased overall cancer risk: A prospective cohort study in Korea. Br. J. Nutr..

[39] Zimmerli B., Rhyn P., Zoller O., Schlatter J. (2001). Occurrence of heterocyclic aromatic amines in the Swiss diet: Analytical method, exposure estimation and risk assessment. Food Addit. Contam..

[40] Mortensen A., Aguilar F., Crebelli R., Di Domenico A., Dusemund B., Frutos M.J., Galtier P., Gott D., Gundert-Remy U., EFSA Panel on Food Additives and Nutrient Sources Added to Food (EFSA ANS Panel) (2017). Scientific Opinion on the re-evaluation of potassium nitrite (E 249) and sodium nitrite (E 250) as food additives. EFSA J..

[41] Knize M., Felton J., Sinha R., Rothman N. (2003). Collaborative Study.

[42] Solyakov A., Skog K. (2002). Screening for heterocyclic amines in chicken cooked in various ways. Food Chem. Toxicol..

[43] Norat T., Bingham S., Ferrari P., Slimani N., Jenab M., Mazuir M., Overvad K., Olsen A., Tjønneland A., Clavel F. (2005). Meat, fish, and colorectal cancer risk: The European Prospective Investigation into Cancer and Nutrition. J. Natl. Cancer Inst..

[44] Mortensen A., Aguilar F., Crebelli R., Di Domenico A., Dusemund B., Frutos M., Galtier P., Gott D., Gundert-Remy U., Lambré C. (2017). Re-evaluation of sodium nitrate (E 251) and potassium nitrate (E 252) as food additives. EFSA J..

[45] Agencia Española de Seguridad Alimentaria y Nutrición (AESAN) Acrilamida. http://www.aesan.gob.es/AECOSAN/web/seguridad_alimentaria/subdetalle/acrilamida.htm%0A.

[46] DellaValle C.T., Xiao Q., Yang G., Ou Shu X., Aschebrook-Kilfoy B., Zheng W., Li H.L., Ji B.-T., Rothman N., Chow W.-H. (2014). Dietary nitrate and nitrite intake and risk of colorectal cancer in the Shanghai Women’s Health Study. Int. J. Cancer.

[47] Zhu Y., Wang P.P., Zhao J., Green R., Sun Z., Roebothan B., Squires J., Buehler S., Dicks E., Zhao J. (2014). Dietary *N*-nitroso compounds and risk of colorectal cancer: A case-control study in Newfoundland and Labrador and Ontario, Canada. Br. J. Nutr..

[48] Augustsson K., Skog K., Jägerstad M., Steineck G. (1997). Assessment of the human exposure to heterocyclic amines. Carcinogenesis.

[49] Food and Agriculture Organization (FAO), World Health Organization (WHO) (2006). Food Additives: Evaluation of Certain Food Contaminants: Sixty-Fourth Report of the Joint FAO/WHO Expert Committee on Food Additives.

[50] Agencia Española de Seguridad Alimentaria y Nutrición (AESAN) (2020). Hidrocarburos Aromáticos Policíclicos (HAPs).

[51] Lee J.-S., Han J.-W., Jung M., Lee K.-W., Chung M.-S. (2020). Effects of thawing and frying methods on the formation of acrylamide and polycyclic aromatic hydrocarbons in chicken meat. Foods.

