# Supplementary material for: Pilot Study for the Dietary Assessment of Xenobiotics Derived from Food Processing in an Adult Spanish Sample
Source: Foods. 2022 Feb 5;11(3):470. doi: 10.3390/foods11030470 (PMC8834107; doi:10.3390/foods11030470)
Supplement: Supplementary file 1 [file foods-11-00470-s001.zip › foods-1516748-supplementary.pdf]

Supplementary Table S1. Description of mean daily xenobiotic intake in the study sample, by gender.

Xenobiotic intake	Total (N=70)	Gender	
		Male (N=25)	Female (N=45)
**Heterocyclic amines (ng/day)**			
AαC	0.02 ± 0.09 (7)	0.01 ± 0.03 (2)	0.02 ± 0.11 (5)
IQ	0.14 ± 0.14 (47)	0.13 ± 0.13 (16)	0.14 ± 0.15 (31)
MeIQ	1.58 ± 1.58 (56)	1.59 ± 1.27 (21)	1.57 ± 1.75 (35)
MeIQx	29.48 ± 27.85 (69)	30.13 ± 30.24 (25)	29.14 ± 26.78 (44)
DiMeIQx	8.18 ± 7.96 (65)	8.71 ± 7.51 (23)	7.88 ± 8.27 (42)
PhIP	187.59 ± 257.04 (70)	221.15 ± 326.16 (25)	168.95 ± 211.15 (45)
**Polycyclic aromatic hydrocarbons (µg/day)**			
B(a)P	0.03 ± 0.03 (69)	0.03 ± 0.02 (25)	0.03 ± 0.03 (44)
DiB(a)A	0.07 ± 0.10 (64)	0.11 ± 0.15 (24)	0.04 ± 0.06 (40)*
Total PAHs	5.04 ± 3.84 (70)	5.01 ± 3.99 (25)	5.06 ± 3.81 (45)
**Nitrates, nitrites and nitroso compounds**			
Nitrates (mg/day)	126.01 ± 97.86 (70)	101.58 ± 73.40 (25)	139.59 ± 107.47 (45)
Nitrites (mg/day)	3.14 ± 2.90 (70)	2.67 ± 1.64 (25)	3.39 ± 3.39 (45)
NDMA (µg/day)	0.17 ± 0.14 (69)	0.17 ± 0.11 (25)	0.17 ± 0.16 (44)
NPIP (µg/day)	0.09 ± 0.09 (67)	0.07 ± 0.05 (25)	0.10 ± 0.11 (42)
NPYR (µg/day)	0.15 ± 0.16 (68)	0.12 ± 0.08 (25)	0.16 ± 0.19 (43)
Comb. (ng/day)	1.71 ± 5.10 (9)	2.40 ± 6.63 (4)	1.33 ± 4.05 (5)
**Acrylamide (µg/day)**	15.12 ± 11.60 (70)	18.36 ± 13.90 (25)	13.33 ± 9.82 (45)

Values are presented as mean ± standard deviation and number of consumers (n). (*) Significant differences were found between genders (p value < 0.05). *T*-test analysis with Bonferroni correction was performed (p value < 0.05). AαC, amino-alpha-carboline; IQ, 2-amino-3-methylimidazo (4,5,f) quinoline; MeIQ, 2-amino-3,4 dimethylimidazo (4,5,f) quinoline; MeIQx, 2-amino-3,8 dimethylimidazo (4,5,f) quinoxaline; DiMeIQx, 2-amino-3,4,8 trimethylimidazo (4,5,f) quinoxaline; PhIP, 2-amino-1-methyl-6-phenylimidazo (4,5,b) pyridine; B(a)P, benzo (a) pyrene; DiB(a)A, dibenzo (a) anthracene; Total PAHs, Total polycyclic aromatic hydrocarbons; NDMA, N-nitrosodimethylamine; NPIP, N-Nitrosopiperidine; NPYR, N-Nitrosopyrrolidine; Comb., Combined nitroso compounds.
